# Rosa26-GFP Direct Repeat (RaDR-GFP) Mice Reveal Tissue- and Age-Dependence of Homologous Recombination in Mammals *In Vivo*

**DOI:** 10.1371/journal.pgen.1004299

**Published:** 2014-06-05

**Authors:** Michelle R. Sukup-Jackson, Orsolya Kiraly, Jennifer E. Kay, Li Na, Elizabeth A. Rowland, Kelly E. Winther, Danielle N. Chow, Takafumi Kimoto, Tetsuya Matsuguchi, Vidya S. Jonnalagadda, Vilena I. Maklakova, Vijay R. Singh, Dushan N. Wadduwage, Jagath Rajapakse, Peter T. C. So, Lara S. Collier, Bevin P. Engelward

**Affiliations:** 1 Department of Biological Engineering, Massachusetts Institute of Technology, Cambridge, Massachusetts, United States of America; 2 Singapore-MIT Alliance for Research and Technology (SMART) Centre, Singapore; 3 School of Pharmacy, University of Wisconsin-Madison, Madison, Wisconsin, United States of America; 4 Department of Mechanical Engineering, Massachusetts Institute of Technology, Cambridge, Massachusetts, United States of America; St Jude Children's Research Hospital, United States of America

## Abstract

Homologous recombination (HR) is critical for the repair of double strand breaks and broken replication forks. Although HR is mostly error free, inherent or environmental conditions that either suppress or induce HR cause genomic instability. Despite its importance in carcinogenesis, due to limitations in our ability to detect HR *in vivo*, little is known about HR in mammalian tissues. Here, we describe a mouse model in which a direct repeat HR substrate is targeted to the ubiquitously expressed *Rosa26* locus. In the *Rosa26* Direct Repeat-GFP (RaDR-GFP) mice, HR between two truncated *EGFP* expression cassettes can yield a fluorescent signal. In-house image analysis software provides a rapid method for quantifying recombination events within intact tissues, and the frequency of recombinant cells can be evaluated by flow cytometry. A comparison among 11 tissues shows that the frequency of recombinant cells varies by more than two orders of magnitude among tissues, wherein HR in the brain is the lowest. Additionally, *de novo* recombination events accumulate with age in the colon, showing that this mouse model can be used to study the impact of chronic exposures on genomic stability. Exposure to N-methyl-N-nitrosourea, an alkylating agent similar to the cancer chemotherapeutic temozolomide, shows that the colon, liver and pancreas are susceptible to DNA damage-induced HR. Finally, histological analysis of the underlying cell types reveals that pancreatic acinar cells and liver hepatocytes undergo HR and also that HR can be specifically detected in colonic somatic stem cells. Taken together, the RaDR-GFP mouse model provides new understanding of how tissue and age impact susceptibility to HR, and enables future studies of genetic, environmental and physiological factors that modulate HR in mammals.

## Author Summary

Cancer is a disease of the genome, caused by accumulated genetic changes, such as point mutations and large-scale sequence rearrangements. Homologous recombination (HR) is a critical DNA repair pathway. While generally accurate, HR between misaligned sequences or between homologous chromosomes can lead to insertions, deletions, and loss of heterozygosity, all of which are known to promote cancer. Indeed, most cancers harbor sequence changes caused by HR, and genetic and environmental conditions that induce or suppress HR are often carcinogenic. To enable studies of HR *in vivo*, we created the *Rosa26* Direct Repeat-Green Fluorescent Protein (RaDR-GFP) mice that carry an integrated transgenic recombination reporter targeted to the ubiquitously expressed *Rosa26* locus. Being able to detect recombinant cells by fluorescence reveals that the frequency of recombination is highly variable among tissues. Furthermore, new recombination events accumulate over time, which contributes to our understanding of why our risk for cancer increases with age. This mouse model provides new understanding of this important DNA repair pathway *in vivo*, and also enables future studies of genetic, environmental and physiological factors that impact the risk of HR-induced sequence rearrangements *in vivo*.

## Introduction

DNA is constantly subjected to endogenous and environmental DNA damaging agents that can lead to toxicity, mutations, and ultimately disease [1]. Maintaining genomic stability in the face of the thousands of DNA lesions that are formed in each cell every day poses a major challenge, especially in the case of double strand breaks (DSBs), which are acutely toxic and can lead to the loss of millions of base pairs if a portion of a chromosome is lost [1], [2]. The two major pathways used by cells to repair DSBs are non-homologous end-joining (NHEJ), which directly rejoins DNA ends, and homologous recombination (HR), which requires a homologous duplex for DSB repair [3]–[8]. The correct balance of NHEJ and HR is essential for preventing genomic instability [4], [9]. If there is a deficiency in HR (*e.g.*, loss of function of *BRCA2*), cells can suffer misrepair of DSBs, resulting in cytotoxicity and translocations that promote cancer and aging [10]–[12]. Ironically, despite the fact that HR is essential, too much HR can also be detrimental, since HR carries the risk of misalignments that cause insertions, deletions, as well as loss of heterozygosity (LOH) [13], [14]. It is likely that HR events contribute to sequence changes in virtually all cancers, since loss of function of almost all tumor suppressor genes requires LOH, and many, if not most, LOH events are caused by HR [14]–[16]. Further, sequence changes generated by HR have been found in multiple cancers [17]–[22], and many conditions that promote HR also promote cancer (as a few examples, exposure to UV light [23], [24], exposure to benzo[*a*]pyrene [25], [26] and mutations in *BLM* [27] and *Ku70/80* [28], [29]).

Dozens of genes are either directly involved in HR or modulate HR activity [6], [30]. An essential early step in HR is the resection of double strand ends to create a 3′ single stranded overhang [31], [32]. Subsequently, BRCA2 helps to load RAD51 onto the single stranded DNA to form a nucleoprotein filament that is capable of homology searching [33]–[37]. Strand invasion leads to formation of a D-loop that is then either resolved by synthesis-dependent strand annealing, which is not associated with crossovers, or by second-end capture and formation of a double Holliday junction, which may or may not be associated with a crossover [5], [30], [38]–[40]. Although crossovers during HR are relatively rare [4], [41], HR-associated crossovers have been shown to cause LOH [14]–[16], [20]. In addition to its important role in the repair of two-ended double strand breaks, HR is essential for repair of one-ended double strand breaks that arise as a consequence of replication fork breakdown [5], [30], [42]. In HR deficient cells, such broken ends cannot be faithfully repaired via reinsertion into the sister chromatid, leading to an increase in misrepair via joining to an inappropriate end [4], [9], [30]. Despite HR's critical role in maintaining genomic stability, little or nothing is known about HR activity in most tissues *in vivo*, due to the lack of effective tools for studying HR in mammals.

Using mouse models that harbor sequences amenable to studies of HR, key insights about HR *in vivo* have been gleaned for certain cells types and tissues. In pioneering work by the Schiestl laboratory, *p* ^un^ mice, which carry a natural duplication wherein a change in pigmentation indicates an HR event, have been used to study the impact of genes and exposures on HR [43], [44]. Additionally, mice engineered to be heterozygous at the *Aprt* locus have been used to show that LOH is often driven by HR *in vivo* [45], [46]. More recently, our laboratory set out to create mouse models in which HR can be detected via direct repeat HR reporters.

**Figure 1:**
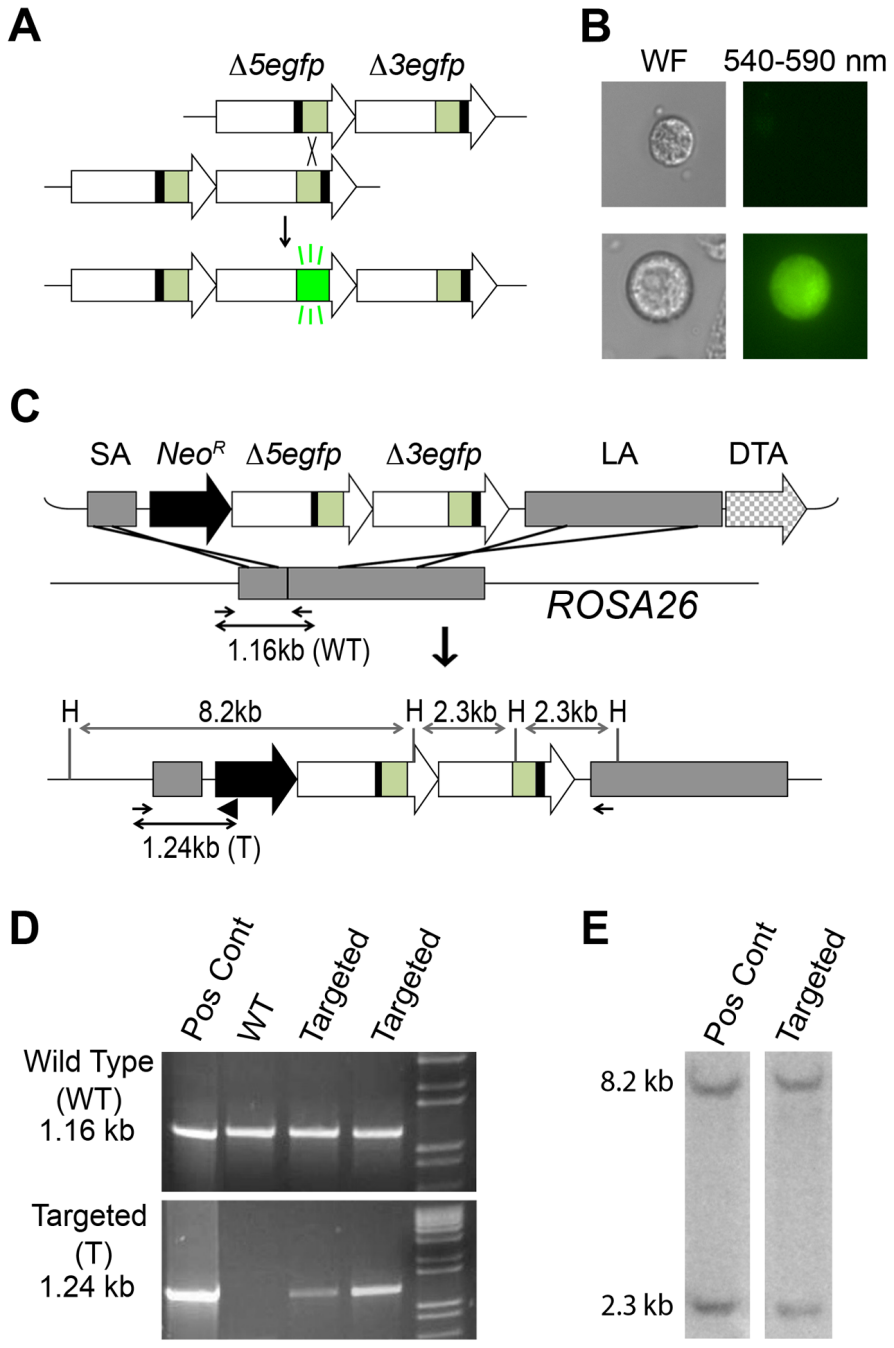
Targeted integration of the RaDR-GFP HR substrate. (A) The RaDR-GFP HR substrate consists of two *EGFP* expression cassettes arranged in tandem (large arrows), each of which is missing essential sequences: deletions at the 5′ (Δ5) and 3′ (Δ3) ends of the coding sequences are indicated by black bars. Coding sequences are in green, and the CAG promoter and polyadenylation (pA) signal sequences are in white. (B) Most cells harboring the RaDR-GFP substrate are non-fluorescent (top) while rare HR events give rise to fluorescent cells (bottom). (C) The RaDR-GFP targeting vector (top) is comprised of a Rosa26 short arm (SA), a positive selection cassette (*Neo* ^R^), the GFP direct repeat HR substrate (described in A), a long arm (LA) and the diphtheria toxin fragment A (DTA) negative selection cassette. Targeted integration gives rise to an 8.2 and 2.3 kb *Hind*III (H) fragment. PCR primers (small arrows) amplify the wild type genomic DNA (1.16 kb) whereas the targeted allele is amplified when a third primer (black triangle) is opposed to the forward primer to give rise to a 1.24 kb product. (D) PCR analysis of a positive control clone, wild type cells and two examples of targeted clones. (E) *Hind*III digested genomic DNA probed with the *EGFP* cDNA reveals 8.2 and 2.3 kb fragments specific to correctly targeted clones.

Studies in *S. cerevisiae* first demonstrated that direct repeat substrates are useful for studying HR [47]–[49]. Briefly, two expression cassettes for a selectable marker are integrated into the genome adjacent to each other. Each expression cassette lacks sequences that are essential for expression. If the expression cassettes misalign and undergo homologous recombination, sequence information can be transferred from one cassette to the other, which can reconstitute full-length sequence to enable expression of the selectable marker (*e.g.*, Figure 1A; black bars indicate deleted sequences). Studies exploiting direct repeat HR substrates in mammalian cells have given rise to fundamental information about the mechanism of HR as well as the impact of sequence orientation, distance between repeats, and exposures on HR [50]–[53]. The Nickoloff laboratory incorporated a site for the homing endonuclease *I-Sce*I, which creates a double strand break that induces HR. Controlling the position of the double strand break gave rise to additional insights into the underlying mechanisms of HR [54], [55]. More recently, the Jasin laboratory designed HR substrates wherein a site-specific double strand break induces HR events that can be detected by expression of EGFP [56], and these assays have been used extensively to reveal the genetic underpinnings of HR [4]. We later created a plasmid-based fluorescence recombination assay which was used for studies of the impact of inflammatory chemicals on HR [57]. To move from *in vitro* studies to *in vivo* studies, we subsequently used elements of the plasmid assay to create a fluorescence-based direct repeat HR substrate in mice. The fluorescent yellow direct repeat (FYDR) mice carry a direct repeat substrate wherein HR can lead to the reconstitution of the full-length coding sequence of the enhanced yellow fluorescent protein (*EYFP*) gene [58], [59]. The FYDR mice are the first genetically engineered animal model that specifically detects HR, and the FYDR HR substrate intentionally does not include a site for artificial introduction of a double strand break (*e.g.*, via *I-Sce*I), since our primary objective is to enable studies of environmental, genetic and physiological factors that modulate HR.

The use of fluorescence has proved to be an effective approach for detecting HR in the FYDR mice *in vivo* [58], [60]–[63]. As expected [50], spontaneous recombination at the HR substrate is rare (the frequency of recombinant cells is ∼1/10^5^) [58], [59]. Nevertheless, the frequency of recombinant cells can be quantified by flow cytometry, and a fluorescent readout makes it possible to identify the cell types that have undergone HR within intact tissue via histological analysis. Furthermore, independent recombination events (as opposed to frequency of cells harboring recombinant DNA) are detectable as fluorescent foci in freshly excised intact tissue by imaging whole organs [60], [61]. To learn more about the factors that impact the frequency of recombinant cells, we also developed a 3D imaging platform for intact tissue, which made it possible to determine how many recombinant cells result from *de novo* recombination events versus cell division [64]. These studies showed that both *de novo* recombination and clonal expansion drive the accumulation of recombinant cells with age [61], [64]. Taken together, studies using the FYDR mice show that fluorescence detection of HR *in vivo* provides valuable insights into genetic, environmental and physiological factors that modulate HR [58]–[60], [62], [63]. Importantly, however, only a limited number of tissues can be studied in the FYDR mice as a consequence of poor expression in some tissues (presumably due to the random locus integration following pronuclear injection) [58], [65]. We therefore set out to generate a recombination reporter mouse with broad reporter expression.

In order to create a mouse model in which HR can be studied in virtually any cell type, we created targeting vectors to enable integration of a direct repeat recombination reporter into the *Rosa26* locus [66]. Here we describe the *Rosa26* Direct Repeat-Green Fluorescent Protein (RaDR-GFP) mice, which harbor two uniquely truncated *EGFP* expression cassettes in tandem. HR at the direct repeat can reconstitute full-length *EGFP* coding sequence, giving rise to fluorescence (Figure 1A). Using this system, we were able to quantify HR in all tissues tested using flow cytometry. Furthermore, we show that several tissues are susceptible to DNA damage-induced HR, and using a novel automated image analysis program for analysis of fluorescence within intact tissue, we show that HR events accumulate in the somatic stem cells of the colon. The RaDR-GFP mice therefore open doors to studies of exposure-induced HR and make it possible to perform an integrated analysis of how cell type, tissue type and age impact HR *in vivo*. Together with the development of quantitative approaches for assessing HR, the RaDR-GFP mice enable studies of how genetic and environmental factors modulate susceptibility to HR events in cancer-relevant tissues.

## Results

### Creation of the RaDR-GFP Mouse

To study recombination *in vivo*, we previously created a direct repeat substrate in which two *EGFP* expression cassettes are positioned in tandem (Figure 1A) [66]. Essential sequences were deleted from each of the *EGFP* cassettes to create *Δ5egfp*, which lacks 15 bp at the 5′ end, and *Δ3egfp*, which lacks 81 bp at the 3′ end. Recombination between the non-functional expression cassettes can reconstitute full-length coding sequence, which can then be expressed under the CMV enhancer/chicken beta-actin promoter [CAG] (Figure 1B) [66], [67]. The promoter, intron, and polyadenylation signal sequences are the same as for the established FYDR mouse model [58]. In the FYDR model, expression levels were high in some tissues (such as pancreas), but there was almost no expression in other tissues (such as the colon), presumably as a consequence of gene silencing associated with the locus of integration.

To enable broad expression, we targeted the HR reporter to the *Rosa26* locus, which was originally identified for its nearly ubiquitous expression [68]. Using a *Rosa26* targeting construct (a kind gift from Dr. P. Soriano) [68], we previously created a targeting vector that includes a short arm (SA), a positive selection marker (*Neo* ^R^), a direct repeat HR substrate, a long arm (LA), and a negative selection cassette (diphtheria toxin fragment A; DTA) (Figure 1C) [66]. The construct design strategy is shown in Figure S1. While our prior studies were focused on HR in ES cells *in vitro*, here we set out to create a knock-in mouse. The targeting construct was electroporated into mouse 129S4/SvJae (129 background) ES cells. Out of 100 colonies, we identified seven candidates using primers designed to yield a 1.16 kb product from wild type DNA and a 1.24 kb product from the targeted allele (Figure 1C–D). Five out of seven candidates harbored the diagnostic 8.2 and a 2.3 kb *Hind*III fragments when analyzed by Southern blot (Figure 1C and 1E). Ten to fourteen 129 ES cells were injected into 3.5-day-old C57BL/6 blastocysts, and the resultant chimeric males were bred with 129 females to establish the RaDR-GFP mouse line. While the 129 background was maintained, the transgene was also backcrossed into the C57BL/6 background for 10 generations. The transgene follows Mendelian inheritance with 49.5% of offspring of heterozygous/wild type parents inheriting the transgene (n = 99).

### Expression of *EGFP* in RaDR-GFP Mouse Fibroblasts Is Caused Specifically by HR

**Figure 2:**
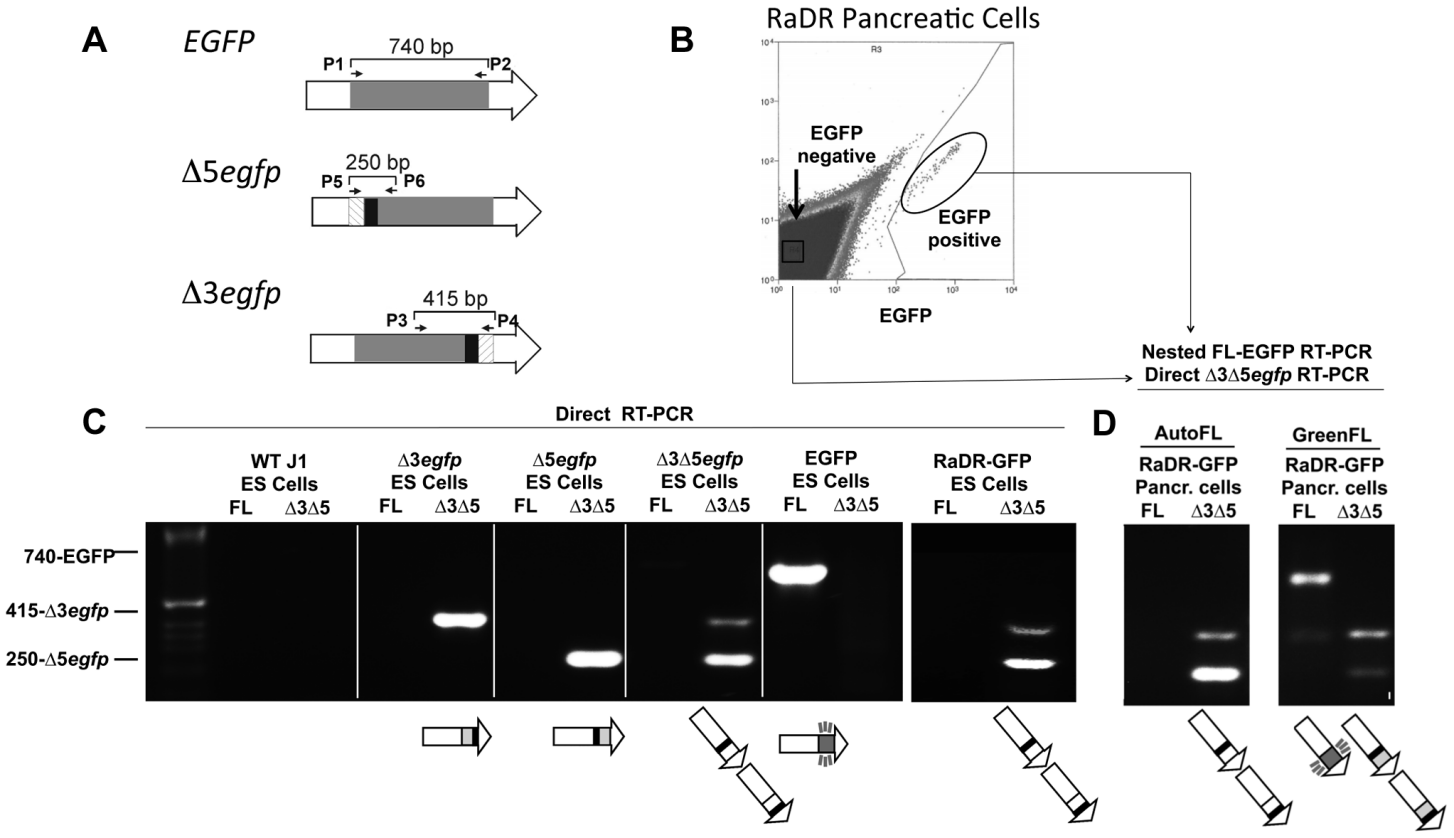
HR leads to reconstitution of full-length *EGFP* coding sequence within green fluorescent RaDR-GFP pancreatic cells. (A) PCR primers (P1–P6) that specifically amplify full length *EGFP*, Δ3*egfp*, and Δ5*egfp* yield the indicated sized fragments (see [66]). Hatched regions indicate unique sequences inserted at the site of the deletions enabling the design of cassette specific primers. (B) Relative fluorescence intensity for 515–545 nm (y axis) and 562–588 nm (x axis), respectively. Expression of *EGFP* leads to a shift to the right. Bracket is drawn to capture the majority of the green fluorescent EGFP positive cells, while excluding autofluorescent cells. (C) PCR analysis using primers that specifically amplify Δ3*egfp*, Δ5*egfp*, and full length *EGFP* to yield a 415, 250 and 740 bp product, respectively. Products are not observed in WT cells (left panel; ladder in lane 1). PCR analysis of targeted clones that each harbor the indicated cassettes demonstrates the specificity of the PCR conditions for each cassette. ES cells used to create the RaDR-GFP mice harbor the Δ3*egfp* and Δ5*egfp* cassettes, consistent with the presence of the unrecombined HR substrate. (D) Fluorescence activated cell sorting and PCR of autofluorescent and green fluorescent pancreatic cells from RaDR-GFP mice reveals the presence of the Δ3*egfp* and Δ5*egfp* cassettes (from the unrecombined HR substrate). Full length *EGFP* coding sequence is uniquely present in the population of green fluorescent cells, consistent with reconstitution of full-length *EGFP* sequence following HR.

To initiate studies of HR in the RaDR-GFP mice, we first analyzed primary ear fibroblasts. Cells were harvested, expanded in culture, and examined by flow cytometry. Gates defining ‘green fluorescent’ and ‘autofluorescent’ cells were drawn conservatively to prevent autofluorescent from being identified as fluorescent, while capturing the majority of the *EGFP* expressing cells (Figure 2B).

To formally determine whether or not green fluorescent cells had indeed undergone HR, we isolated fluorescent cells to learn if they harbor full-length *EGFP* coding sequence. We previously designed PCR primers that specifically amplify Δ3*egfp*, Δ5*egfp*, or full-length *EGFP* (Figure 2A and Table S1). Here, we developed methods to analyze cells for the presence or absence of each cassette using cDNA as a template, rather than genomic DNA as previously described [66]. Our rationale for this approach was that by exploiting the multiple copies of cassette sequences present in mRNA, we would be able to query the presence and absence of cassettes in single cells in future experiments. As a first step, primers were used to analyze cDNA from control ES cell lines that had previously been targeted with each cassette individually, as well as ES cells that harbor both Δ3*egfp* and Δ5*egfp* [66]. Conditions were optimized so that both Δ3*egfp* and Δ5*egfp* are detectable in a single PCR reaction so that each cassette serves as a positive control for the other. Results show specific detection of each cassette in isolation and together, and full length sequence is only observed in the positive control *EGFP* expressing cells, as expected (Figure 2C, first five panels). To create the RaDR-GFP mice, we created new early passage clones of ES cells targeted with the recombination substrate. PCR analysis of RaDR-GFP cells that carry the unrecombined substrate reveals both the Δ3*egfp* and Δ5*egfp* cassettes, but not the full length *EGFP*, as expected (Figure 2C, panel six).

Having created RaDR-GFP mice that carry the *Rosa26* targeted HR substrate (Figure 1C–E), we next set out to determine whether or not fluorescent cells from these animals indeed harbor the full length *EGFP* sequence, as anticipated following HR. Fluorescent and autofluorescent control cells were isolated from a single cell suspension of disaggregated RaDR-GFP pancreatic cells using FACS (Figure 2B). Primers that flank the coding sequence were optimized for nested PCR (Table S2), and cDNA was analyzed either by direct PCR or nested PCR, as indicated. Analysis of autofluorescent RaDR-GFP pancreatic cells revealed the presence of Δ3*egfp* and Δ5*egfp*, whereas full-length *EGFP* sequence was not detected (Figure 2D). In contrast, full-length *EGFP* was readily detected in samples of green fluorescent RaDR-GFP pancreatic cells (Figure 2D). The Δ3*egfp* and Δ5*egfp* cassettes were also detected (Figure 2D), which is consistent with their potential retention following HR (Figure 1A). The RaDR-GFP HR substrate is designed so that over a dozen base pairs need to be restored to give rise to a functional full-length *EGFP* coding sequence [66]. As restoration of a significant number of nucleotides requires HR for alignment and transfer of sequence information, these data show that fluorescence is an indicator of homologous recombination at the RaDR-GFP substrate.

**Figure 3:**
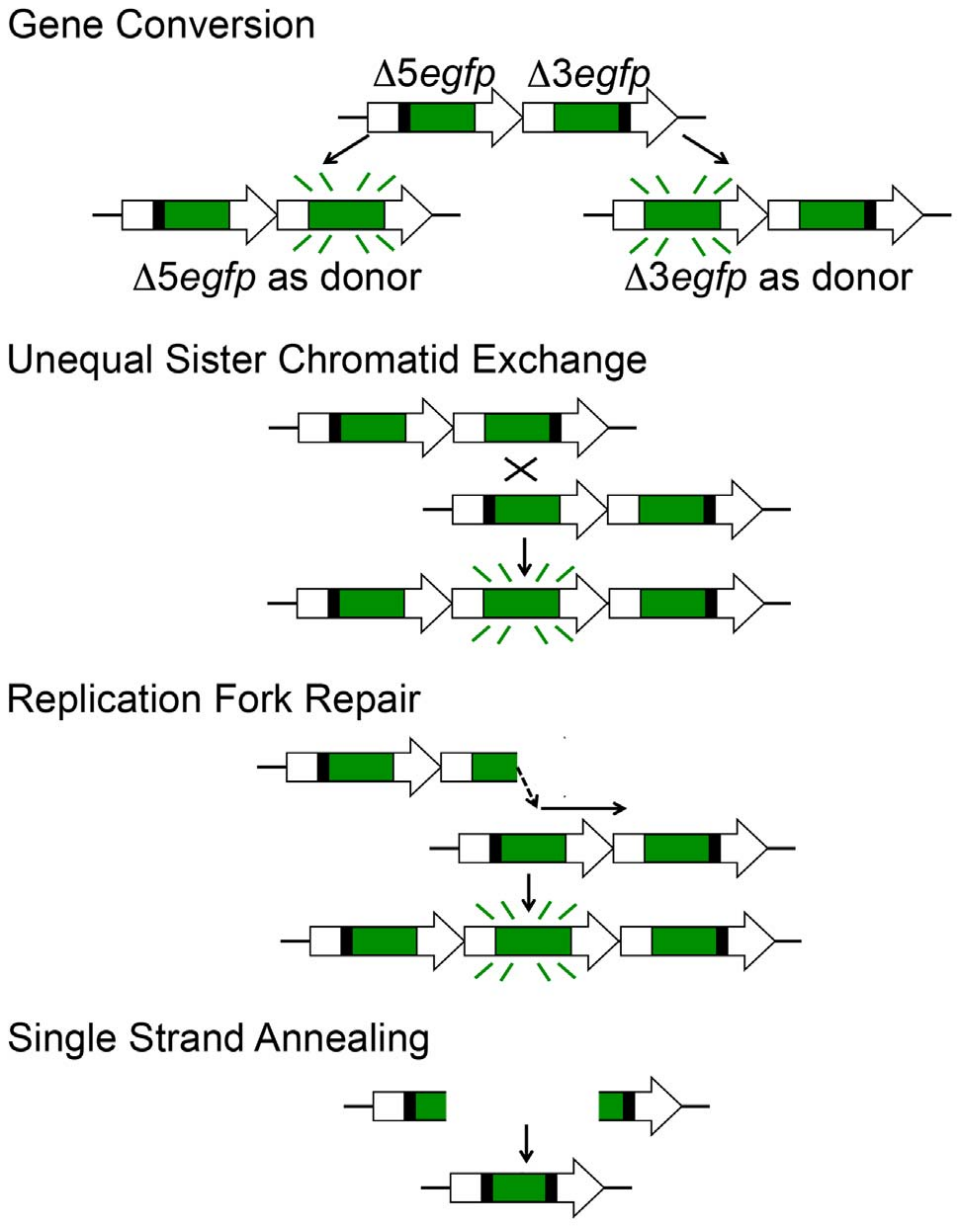
HR at the RaDR-GFP substrate can give rise to fluorescence following gene conversion, sister chromatid exchange, and replication fork repair, but not following SSA. Each cassette is missing different essential coding sequences such that neither is able to express EGFP. Gene conversion can lead to transfer of sequence information from one cassette to the other, restoring full-length *EGFP* coding sequence and giving rise to a fluorescent readout. Each cassette can be the donor or the recipient in a gene conversion event. The entire HR reporter is copied during S phase, making it possible for crossovers between sister chromatids (gene conversion with crossover) to reconstitute full-length *EGFP*. Note that a long tract gene conversion event would be indistinguishable. Recombination that arises as a consequence of repair of a broken replication fork can also be detected using the RaDR-GFP substrate. A replication fork breakdown arising from a fork moving from left to right is shown. Reinsertion of the broken Δ3*egfp* end into the Δ5*egfp* cassette can restore full length EGFP. Note that this figure depicts events wherein the replication fork had been moving from left to right; *EGFP* can analogously be restored by repair of forks moving in the opposite direction (not shown). Single strand annealing initiated by a DSB between the repeated cassettes can be readily repaired, but these events will not reconstitute full-length EGFP and thus SSA cannot be detected.

Ultimately, this mouse model can be used to study the underlying molecular changes that caused sequences to be restored to full length. Gene conversions without a crossover can be identified by the presence of one of the two original cassettes, along with full-length sequence. In contrast, replication fork repair or gene conversion with crossover will result in a triplication wherein both of the original cassettes are present along with the full-length sequence (Figure 3). We had previously performed this type of analysis on ES cells that had been clonally expanded *in vitro* [66]. Here, we set out to develop methods that would enable studies of HR *in vivo*. Because clonally expanding single cells from mouse tissues is difficult, we set out to develop methods that would enable analysis of single fluorescent cells isolated from mouse tissues using FACS. Initial data indicate that single cell analysis can indeed be used to identify cells with each of the three major recombination classes (Figure S2B).

### Positive Control Mice Reveal Broad Expression of *EGFP In Vivo*

**Figure 4:**
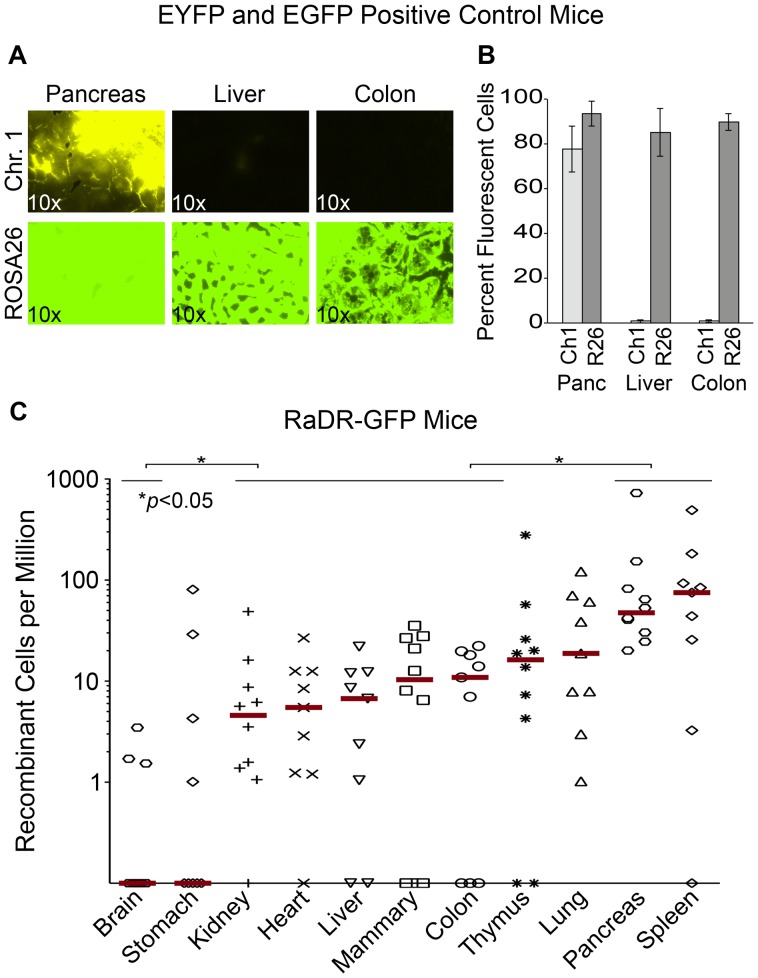
Analysis of EYFP and EGFP positive control mice and RaDR-GFP tissues. (A) Histological images of FYDR positive control mice that harbor full-length *EYFP* sequences within mouse Ch. 1, and RaDR-GFP positive control mice that harbor full-length *EGFP* at the *Rosa*26 locus expressed under the same CAG promoter (see Materials and Methods). Brightness/contrast for EYFP filtered images (×10) was adjusted equivalently for all images. (B) Quantification of percentage of cells that are fluorescent within disaggregated pancreas, liver and colon of the FYDR and RaDR-GFP positive control mice (measured using flow cytometry). Almost no cells are fluorescent in liver and colon cells from the positive control FYDR mice, indicating that these tissues cannot be used for analysis of HR in the FYDR mice. Almost all cells from the pancreas, liver and colon of the RaDR-GFP positive control mice are fluorescent, indicating that these tissues can be analyzed for HR frequency in the RaDR-GFP mice. (C) Frequency of HR among 11 different tissues from two months old RaDR-GFP mice is highly variable. The number of recombinant cells per million is reported as individual data points (one data point for each mouse; samples from 9–10 mice were analyzed for each type of tissue). Horizontal lines that capture more than one tissue type indicate that samples within that group are not statistically significantly different from one another. Statistically significant differences between groups (of one or more tissue types) are noted. Bars indicate median frequencies.

Previous studies of FYDR positive control mice (which express *EYFP* from the same promoter and locus as the HR reporter) show that there is little or no expression of *EYFP* in many tissues (presumably due to silencing), which greatly limits the utility of the FYDR model [65]. While we anticipated that targeting the *EGFP* direct repeat reporter to a site with ubiquitous expression would overcome this barrier to studies of HR, prior studies of expression at the *Rosa26* locus had been done using the *Rosa26* promoter [68], whereas the CAG promoter drives the RaDR-GFP transgene. To address the formal possibility that *EGFP* expression from the RaDR-GFP reporter might not be ubiquitous, we assessed the extent of expression of *EGFP* from a positive control mouse in which *EGFP* is expressed specifically from the CAG promoter at the *Rosa26* locus (see Materials and Methods for details). Analysis of tissues from the FYDR positive controls showed high expression of *EYFP* in the pancreas, and low expression in the liver and the colon (Figure 4A, upper row), which is similar to the low expression previously observed in the kidney and lung [65]. In contrast, expression of *EGFP* in the *Rosa26* positive control mice was very strong in all three tissues (Figure 4A, bottom row). By using the same imaging parameters, these data also show that fluorescence from EGFP is significantly brighter than that of EYFP. Analysis by flow cytometry similarly shows that EGFP fluorescence is high not only in pancreas, liver and colon (Figure 4B), but also in eight additional tissues (Table 1). The nearly ubiquitous expression of *EGFP* in the positive control mice suggests that fluorescent recombinant cells in the RaDR-GFP mice would be detectable in most mouse tissues. Furthermore, the positive control mice are essential for comparisons of HR frequency among tissues, since the frequency of GFP positive cells in the positive control mice provides the required baseline for comparing HR frequencies among tissues in the RaDR-GFP mouse model.

**Table 1:** Percentage of fluorescent cells in disaggregated RaDR-GFP tissues.

Tissue	Fluorescent (%)
Brain	91±6
Breast	87±9
Colon/Cecum	90±4
Heart	74±18
Kidney	79±9
Liver	86±10
Lung	79±18
Pancreas	94±5
Spleen	83±2
Stomach	75±24
Thymus	83±13

Tissue was disaggregated and analyzed by flow cytometry. Gating to capture EGFP positive cells was set to stringently exclude autofluorescent cells from wild type mice (see Materials and Methods for details).

### HR Is Detected in 11 Major Organs and Tissues

To explore the feasibility of studying HR in multiple tissues (including tissues that had previously been inaccessible to HR analysis), 11 tissues from RaDR-GFP mice were disaggregated and analyzed by flow cytometry, first by gating for live cells, and subsequently by gating for fluorescent cells. Remarkably, fluorescent recombinant cells were present in all tissues (Figure 4C). Recombinant cells were relatively frequent in the pancreas (similar to the FYDR mice) and in the spleen. Recombinant cells were also observed at a significant frequency in the kidney, heart, liver, mammary gland, and colon of the RaDR-GFP mice (all of which had previously been inaccessible for studies of HR within mammalian tissues *in vivo*). In contrast, very few fluorescent cells were detected in stomach or brain tissue (Figure 4C). The observation that ∼90% of cells from brain tissue of the *Rosa26* positive control mice are fluorescent (Table 1) indicates that fluorescent recombinant cells can be detected. These results together therefore show that there are very few recombinant cells in the brain (note that the detection of rare fluorescent cells is limited to ∼1/10^6^). One possible explanation for the low frequency of EGFP positive cells in the brain is the short time period during which HR is active in the developing brain [69], where it plays a critical role in neurogenesis and cancer suppression [70]. It is possible that relatively few recombinant cells accumulate in the RaDR mouse brain compared to other tissues due to the short time during which HR is highly active. Although further studies are needed for a more in depth understanding of HR among tissues, taken together, these studies show for the first time that spontaneous HR is pervasive in adult mammalian tissues.

### Visualization of Recombinant Cells within Intact Tissues Enables Quantification of HR Events

**Figure 5:**
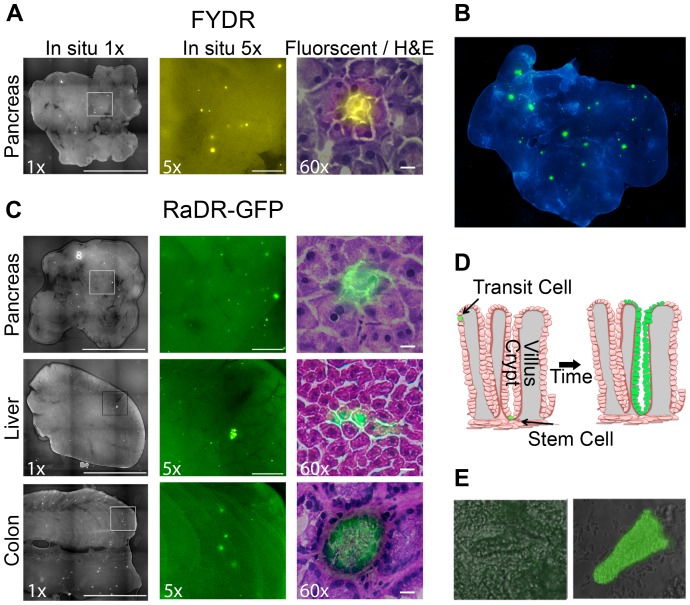
Fluorescence detection of recombinant cells within intact tissues of FYDR and RaDR-GFP mice and identification of the underlying cell types. (A) Analysis of pancreatic tissue from FYDR mice. Foci can be detected within images of the entire organ compressed to 0.5 mm (left image is at ×1, scale bar = 1 cm). Foci are readily quantifiable at ×5 (middle image, scale bar = 1 mm). Histological image of H&E stained section (right image at ×60, scale bar = 20 µm) overlaid with fluorescence image (510–560 nm filter). Brightness/contrast for fluorescent images was optimized for each histological section. Fluorescence is pseudocolored. (B) Analysis of pancreatic tissue (nearly the entire organ) from a RaDR-GFP mouse compressed to 0.5 mm. Nuclei are stained with Hoechst; fluorescent recombinant cells are pseudocolored green. (C) *In situ* detection of recombinant cells within pancreas, liver and colon from RaDR-GFP mice. Image collection was done according to (A). Recombinant pancreatic acinar cells, liver hepatocytes and colonic epithelial cells are quantifiable within freshly excised tissues (left and middle images). Cell types can be discerned using H&E overlay (right images). (D) Crypt model emphasizing that recombinant transit cells are rapidly lost, while recombinant somatic stem cells can give rise to a persistent wholly fluorescent crypt. (E) Analysis of disaggregated crypts reveals the presence of non-fluorescent crypts (left) and crypts in which essentially all of the epithelial cells fluoresce (right).

Our previous studies, as well as results presented here, show that recombinant cells can be detected *in situ* within intact pancreata of FYDR mice as fluorescent foci (Figure 5A) (see [60], [61]). Importantly, since recombination is a rare event and pancreatic cells do not migrate significantly, independent recombination events can be identified as isolated fluorescent foci. Analysis of recombination events provides greater sensitivity compared to the frequency of recombinant cells as a means for detecting genetic and environmental factors that modulate HR [65].

To explore the efficacy of RaDR-GFP mice for studies of HR events within intact tissue, pancreatic tissue from a RaDR-GFP mouse was stained with DAPI and imaged using fluorescence microscopy at low magnification (×1). Fluorescent foci are readily apparent in the RaDR-GFP pancreatic tissue (Figure 5B). Tissue from 11 RaDR-GFP mice was compressed to 0.5 mm and imaged for manual quantification of foci. Using this approach, we observed that the median frequency of spontaneous recombination events is ∼140/cm^2^. In addition, unlike the FYDR mice, recombinant foci are also readily detected in both the intact liver and the intact colon (Figure 5C).

Differences in the frequency of foci among tissues reflect both the frequency of HR events as well as the optical properties of each tissue. Therefore, it is difficult to discern tissue-specific differences in HR using this approach (note that flow cytometry of disaggregated tissues overcomes this limitation). Importantly, however, for studies of factors that modulate HR in a specific tissue, analysis of HR events *in situ* provides a powerful approach both in terms of increased sensitivity [65] and in terms of learning about HR in specific cell types (see below).

### Histological Identification of Recombinant Cell Types

Although HR events are rare, it is nonetheless possible to identify fluorescent foci within frozen 5 µm sections using epifluorescence microscopy. After imaging, sections can be stained with hematoxylin and eosin (H&E) to reveal tissue architecture. Image overlays for pancreatic fluorescent foci reveal that for both FYDR and RaDR-GFP, recombination is detected in pancreatic acinar cells (Figure 5A and 5C, right). These observations are consistent with studies of FYDR mice in which analysis of >100 pancreatic foci revealed only acinar cells [61]. In the case of liver and colon, overlay of fluorescent images with H&E images reveals fluorescent hepatocytes in the liver, and fluorescent epithelial cells in the colon (Figure 5C). Pancreatic acinar cells, liver hepatocytes and colonic epithelial cells all give rise to tumors in their respective tissues, raising the possibility that the RaDR-GFP mice can be used to study the etiology of cancer (see Discussion).

### Detection and Quantification of HR Specifically within Somatic Stem Cells

Somatic stem cells are of particular interest in cancer research. In the colon, there are only one or a few somatic stem cells at the base of each colonic crypt. Somatic stem cells are defined as being cells that have the ability to give rise to the epithelial layer in that crypt [71]–[73]. Therefore, a single HR event in a colonic somatic stem cell can lead to “crypt conversion” wherein all of the epithelial cells of its crypt share the same genetic change (Figure 5D). Since transit cells are short lived, lasting only a few days before the epithelial layer of the crypt is replaced [73], mutations in transit cells are less likely to contribute to cancer compared to mutations in colonic somatic stem cells, which can persist throughout the lifetime of the animal [73].

Analysis of thin sections via epifluorescence microscopy revealed a cross section of a colonic crypt in which it appears that all of the central epithelial cells are fluorescent (Figure 5C, bottom right), suggesting that a stem cell from this crypt replaced the crypt epithelial cell layer with fluorescent daughter cells (crypt boundaries can be identified by a ring of epithelial cells with higher staining intensity; Figure 5C). To learn more about the possibility of crypt conversion, colonic tissue was processed to gently remove crypts. Intact wholly fluorescent crypts were readily identified among disaggregated crypts from RaDR-GFP mice (*e.g.*, Figure 5E), which is consistent with replacement of crypt epithelial cells by a single somatic stem cell that had undergone HR at the RaDR-GFP substrate. Taken together, the RaDR-GFP mice enable studies of HR in a cell type that is highly relevant to colon cancer.

### Recombinant Somatic Stem Cells Accumulate with Age in the Colon

**Figure 6:**
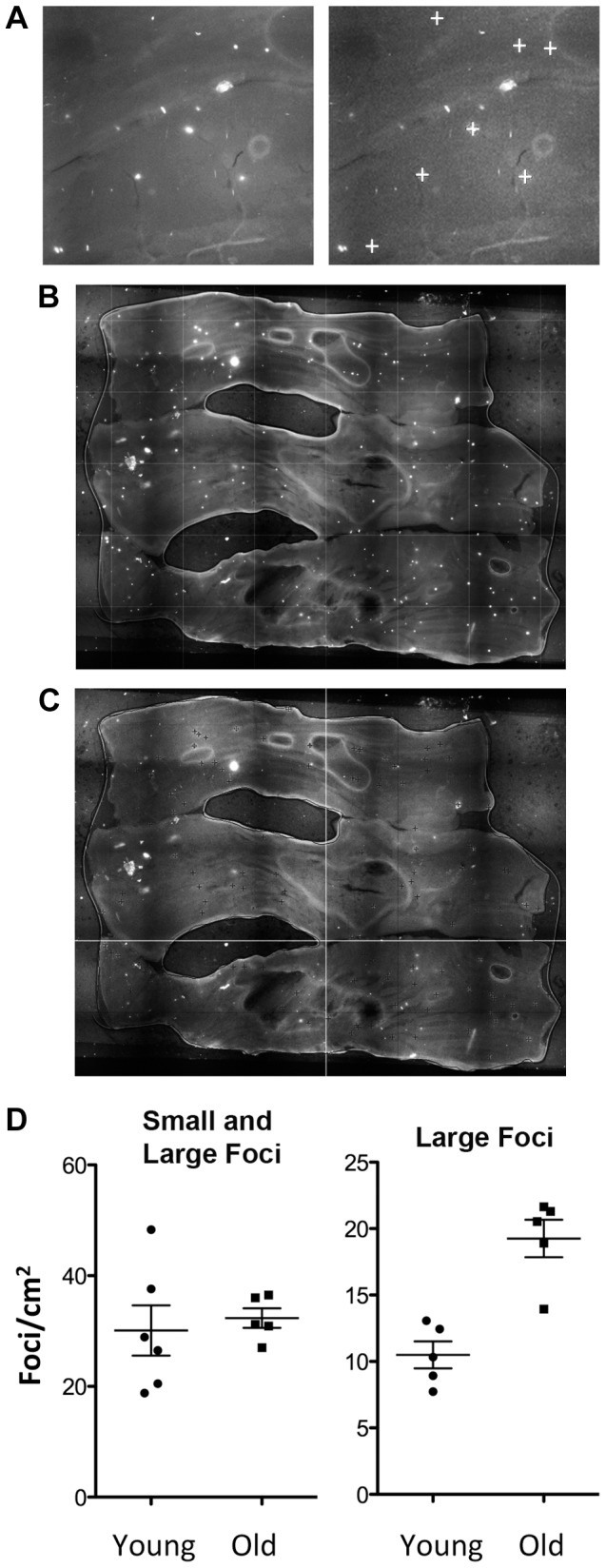
Recombinant cells accumulate with age in the colon. (A) Image analysis with in-house software designed to detect large foci with consistent morphology. Note that small foci and irregularly shaped foci are not designated positive by the program (compare left and right images; “+” symbols indicate foci identified by the program). (B) Freshly excised colonic tissue opened to reveal the lumen is pressed between coverslips and imaged using an epifluorescent microscope. (C) Image analysis using in-house software marks large foci with a dark cross. Comparing B and C shows that most of the large foci (bright white spots) are recognized by the program (dark cross marks). (D) Quantification of recombination events by analysis of foci frequency in the colon. Each symbol indicates the foci frequency for tissue from a single mouse (N = 5–6). The entire surface area was imaged in order to suppress the impact of variation in different regions of each tissue. Images were compiled, and the frequency of foci was determined for the entire organ, which was then divided by the surface area (determined using ImageJ). Each symbol represents the average number of foci/cm^2^ for the entire organ from each animal in cohorts of juvenile and aged animals. Bars indicate medians. Both small and large foci were counted manually (left). The same samples, when analyzed using in-house software that identifies large crypts, shows a statistically significant increase in the aged animals (*p*<0.01, Student's *t*-test) (right). Large foci are consistent with HR in colonic somatic stem cells that lead to wholly fluorescent crypts.

Aging is a critical risk factor for almost all cancers. To learn about the potential for recombinant cells to accumulate with age in the colon, we imaged and analyzed colonic tissue from young (3–4 months old) and old (9–10 months old) animals. Foci were counted by eye in a blinded fashion, and results indicated that there was no significant difference in the frequency of recombinant cell foci between the young and old animals (Figure 6D, left). Foci in colonic tissue appear both as a consequence of transit cell recombination and somatic stem cell recombination. Given that transit cells are only present for a few days, unless the rate of recombination changes for young and old animals, one would not anticipate an observable increase in the frequency of transit cell foci. In contrast, as described above, somatic stem cells can persist for years [73], which raises the possibility that fluorescent foci that result from recombination events in stem cells would accumulate and be detectable by the presence of whole crypt conversion. In order to favor detection of HR events in somatic stem cells, we therefore set out to create an image analysis program that differentiates large foci (more likely to be due to whole crypt conversion) from small foci (more likely to be the result of HR in transit cells).

We created a foci counting program that favors detection of large foci by using automated quantification techniques that exploit both intensity and morphological features. Classification was enabled using support vector machines. We simulated the data using a noise model, which includes the homogenous noise of the sample as well as the detection noise, to analyze the performance of our algorithms. To avoid false positives, only large foci with a consistent morphology and intensity were counted, and small foci or irregularly shaped foci were excluded (Figure 6A). Although this approach has a potentially high false negative frequency, it is more important to avoid false positives than false negatives. Analysis of the lumen of large samples of colonic tissue shows the clear appearance of bright foci (Figure 6B). Using the automated analysis software, large foci were marked with a dark cross if considered to be positive (Figure 6C). Direct comparison of Figure 6B and Figure 6C shows that the majority of the large foci are identified by the program. We validated this approach by comparing the automated counting results to manual counts. A more detailed description of this software will be published separately.

Using our image analysis software, we reanalyzed the colonic tissue from young and old mice. Remarkably, there is a highly significant (*p*<0.01) increase in the frequency of larger foci with age (Figure 6D, right). Since the largest foci result from clonal expansion of somatic stem cells, these results indicate that recombination events indeed accumulate in colonic somatic stem cells. It is noteworthy that inclusion of foci from transit cells is anticipated to lead to smaller foci that mask detection of changes in the more rare larger foci, as indicated in Figure 6D (left) such that inclusion of false positives damps the signal from the somatic stem cells. Taken together, these results provide some of the first insights into the relative susceptibility of transit cells and somatic stem cells to recombination with age, and open doors to future studies of conditions that modulate the risk of recombination in cells that have the potential to give rise to cancer.

### RaDR-GFP Mice Enable Studies of Exposure-Induced HR in Multiple Tissues

**Figure 7:**
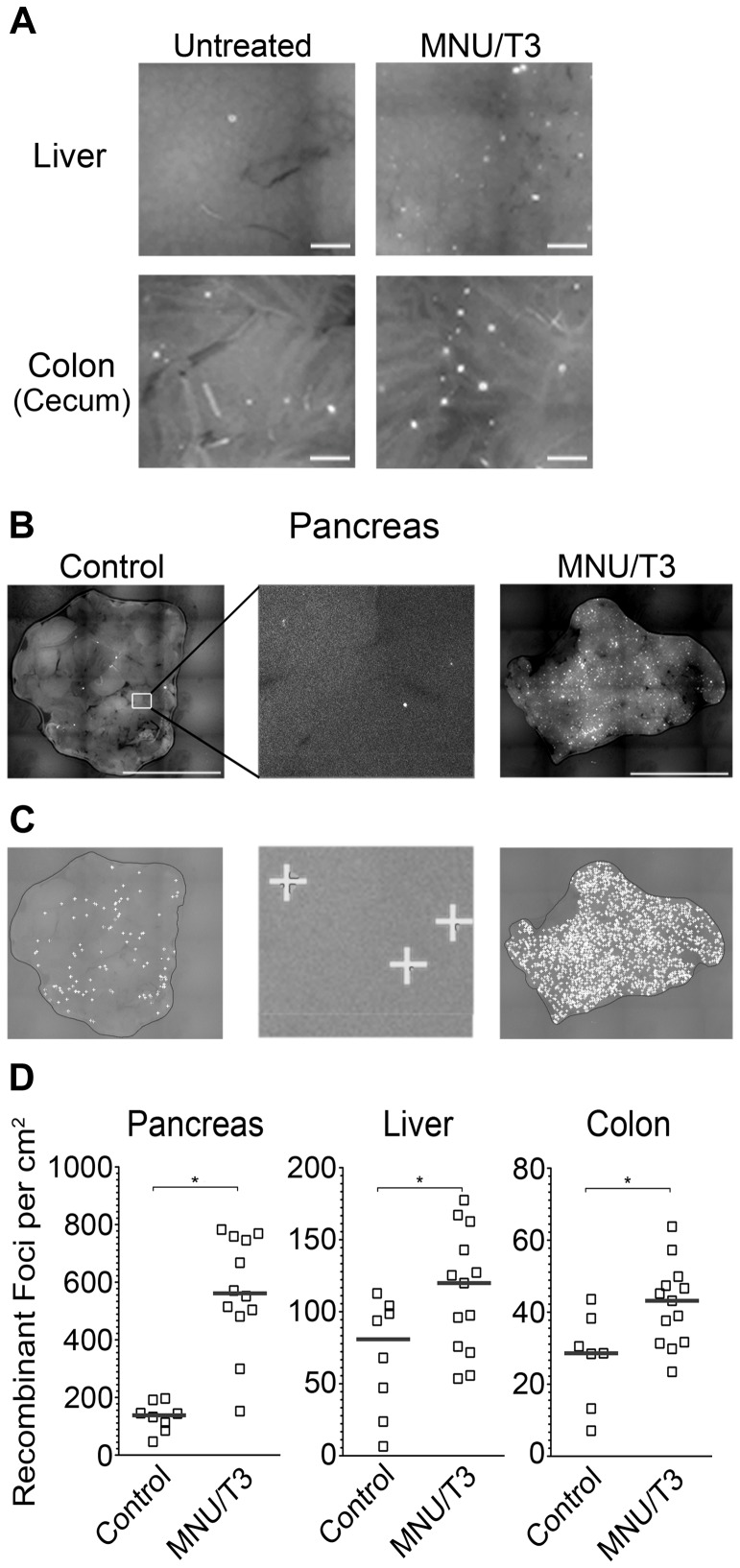
HR events are induced by exposure to an exogenous DNA damaging agent and are quantifiable using in-house software. (A) Images of freshly excised liver and colon tissue from control mice and from mice that were exposed to MNU/T3. (B) Images of pancreata from control and MNU/T3 treated RaDR-GFP mice. (C) Analysis of images from part (B) using in-house software to quantify fluorescent foci. Foci identified by the program are indicated by “+”. (D) Frequencies of recombinant foci per cm^2^ in pancreatic, liver and colon tissue quantified using in-house software (controls N = 7–8; treated N = 12–13). Brightness and contrast for all images were optimized for publication. ^*^ *p*<0.05, Mann–Whitney *U*-test.

Alkylating agents are carcinogenic, used for cancer chemotherapy, and have been shown to be recombinogenic in mice [74], [75]. We were therefore interested in the extent to which RaDR-GFP tissues would be susceptible to exposure-induced HR. Here, we focused on methylnitrosourea (MNU), a model S_N_1 alkylating agent similar to temozolomide, which is used in cancer chemotherapy [74]. In parallel ongoing studies of FYDR mice, we tested multiple exposure conditions for efficacy in inducing HR, and we found that the combination of MNU and thyroid hormone (T3), which impacts pancreas physiology, was the strongest inducer of HR among the conditions that we tested. We therefore asked whether or not the RaDR-GFP model is sensitive to exposure-induced HR by treating animals with combined MNU/T3 (see Materials and Methods). In addition to pancreas, we also evaluated colon and liver (Figure 7A). For all three tissues, MNU/T3 was a strong inducer of HR. For the pancreas, the increase in the frequency of *de novo* recombination events was most dramatic (Figure 7B), making it infeasible to quantify recombinant foci manually. Automated image analysis using a modified version of our foci analysis program (optimized for the pancreas) enables quantification of small/faint foci that are difficult to quantify by eye (Figure 7C). Furthermore, the automated foci counting program enables future studies of foci characterization based on size and other morphological characteristics. Automated quantification of foci in RaDR-GFP mouse pancreata shows that, on average, exposure to MNU/T3 leads to ∼400 new recombination events per cm^2^ (Figure 7D). In addition to the pancreas, exposure-induced HR was also observed in the liver and colon of RaDR-GFP mice (Figure 7D). Taken together, these results demonstrate the efficacy of RaDR-GFP mice for studies of exposure-induced HR in multiple tissues.

## Discussion

Although HR is essential [76], [77], its activity must be carefully controlled in order to maintain genomic integrity [30], [70]. Inherent defects that either suppress or induce HR are known to be tumorigenic [11] and exposures that induce HR are often carcinogenic [22], [44]. Despite its importance, progress in our understanding of the role of HR in mammals has been hampered by the lack of effective tools for studying HR in many mammalian tissues. Here, we describe the RaDR-GFP mice, which harbor an integrated direct repeat that causes cells to fluoresce following HR. By targeting the reporter to the *Rosa26* locus, expression of the transgene is nearly ubiquitous, thus enabling studies of HR in nearly all major organs, including liver, colon, spleen, heart, lung, kidney, stomach, thymus, brain, breast, and pancreas, many of which have been hitherto inaccessible for analysis.

HR events at the RaDR-GFP substrate can occur via several different mechanisms. Prior studies of ES cells show that most recombinant fluorescent RaDR-GFP cells have undergone gene conversion without crossovers [66], which are thought to result primarily from the synthesis dependent strand annealing pathway (see [5], which includes animations for HR pathways). DSB-induced crossovers between sister chromatids can also be detected by the RaDR-GFP substrate. Importantly, one of the critical roles of HR is to repair one-ended DSBs at broken replication forks, and these events can readily be detected using the RaDR-GFP substrate (Figure 3). One challenge when using the direct repeat approach for studies of HR is that these canonical HR events can be overshadowed by single strand annealing (SSA), a subpathway of HR that is the most frequent spontaneous event at a direct repeat [5], [66]. Specifically, when a DSB is formed between repeats, the ends are resected to reveal 3′ overhangs that can readily anneal to one another. As we are primarily interested in conditions that stimulate problems during replication, we designed the RaDR-GFP substrate so that SSA is not detected (Figure 3 shows that SSA gives rise to an expression cassette that harbors both of the original deletions). This approach enables studies of spontaneous and exposure-induced HR events that are less frequent at a direct repeat, yet biologically important, such as replication fork repair. Taken together, both DSBs and broken replication forks can lead to fluorescence in the RaDR-GFP model, thus providing a window into how mammalian cells respond to a broad range of conditions that impact genomic stability by either suppressing or inducing HR *in vivo*.

To learn about spontaneous HR *in vivo*, we quantified recombinant fluorescent cells in 11 different tissues and found that recombinant cells are present in all tissues studied. The frequency of recombinant cells is highly variable among tissues, ranging from very low in the brain and stomach, to very frequent in the pancreas and spleen. The observation that recombinant cells are relatively frequent in the pancreas suggests that HR is highly active in this organ (which is consistent with the studies of aging; see below). Interestingly, mutations in *BRCA2*, which plays a key role in initiating HR, are known to increase the risk of pancreatic cancer [30], [78]. Thus, for the pancreas, there is a correlation between HR activity and the potential for a defect in HR to contribute to cancer [79]. For some other tissues, the frequency of HR is either unexpectedly high, or unexpectedly low. In the case of the heart, which has a relatively low proliferative index, there are a surprisingly high number of recombinant cells. One possibility is that progenitor cells that gave rise to cardiac tissue underwent HR, leading to the appearance of recombinant fluorescent cells in the adult tissue. One way to differentiate HR during development versus in the adult animal is to monitor tissue during aging to see if HR is active in adult animals. In contrast to cardiac tissue, the stomach had an unexpectedly low frequency of recombinant cells. It is noteworthy that not all of the cells in the disaggregated stomach tissue from the positive control mice were fluorescent (∼75% were positive by flow cytometry). This means that for some cell types, HR will not give rise to fluorescence. Although beyond the scope of this particular study, knowledge about HR in specific cell types can be achieved through a comparison of EGFP expression in RaDR-GFP mice (yielding information about HR) and EGFP expression in the positive control mice (yielding the baseline frequency of cells in which HR can be detected).

As the RaDR-GFP mice age, the frequency of recombinant somatic stem cells increases in the colon. Being able to monitor the burden of recombinant cells is valuable for long-term studies of conditions that impact HR. The burden of cells harboring sequence changes is critical to cancer development since an increase in the frequency of cells harboring a tumorigenic mutation leads to an increased risk of subsequent tumor-promoting mutations. Interestingly, exposure to MNU/T3 induced hundreds of recombination events in the RaDR-GFP mice. In essence, the burden of recombinant cells in young DNA damage-exposed mice is similar to aged mice, calling attention to the burden of mutant cells as a commonality for these two key risk factors for cancer. Being able to monitor HR over time and in response to exposures shows that RaDR-GFP mouse model can be used for studies of long-term exposures and physiological factors that impact the burden of recombinant cells, thus providing insights into fundamental processes that promote cancer.

A key advantage of fluorescence as a marker for HR is that it is possible to reveal the underlying cell types that have undergone HR. Using a fluorescent overlay on H&E images, we observed fluorescent recombinant pancreatic acinar cells, liver hepatocytes and colonic epithelial cells. Knowledge about genomic stability in all three of these cell types is relevant to cancer. Although most pancreatic carcinomas are thought to originate from ductal cells [80], mutation of Kras in acinar cells can lead to neoplasia of the ductal phenotype [81], and furthermore there is evidence that acinar cells can undergo acinar to ductal transdifferentiation [82]. HR is also detectable in hepatocytes, which are precursors to hepatocellular carcinomas. Additionally, being able to study genetic change *in vivo* in the liver has broad implications, since liver genotoxicity is a major barrier in drug development [83]–[85]. In the colon, we observed HR in colonic epithelial cells. Most epithelial cells are rapidly sloughed off, making these cells unlikely targets for initiating mutations for cancer. In contrast, colonic somatic stem cells persist for years [72], [73]. Our observation that there are crypts in which all cells appear to be fluorescent is consistent with an HR event in a somatic stem cell or early daughter cell of that crypt. Interestingly, methods have previously been developed for visualizing cells that have lost *Dlb*-1 gene function in colon crypts [86]. In *Dlb-*1 heterozygous mice, LOH can lead to a positive crypt by any of several different mechanisms (*e.g.*, point mutations, frameshifts, deletion, HR, *etc.*). An advantage of the RaDR-GFP substrate is that it is designed to specifically detect HR.

To learn about exposure-driven HR, we elected to exploit an alkylating agent that provides insights into the biology of cancer chemotherapeutics. The model agent MNU is an S_N_1 type methylating agent that generates methylated bases such as 3-methyladenine, 7-methylguanine and *O* ^6^-methylguanine [74]. Several methylating agents creating these lesions are currently used in cancer chemotherapy including temozolomide, which is used to treat metastatic melanoma and malignant gliomas [87]. Importantly, HR activity contributes to resistance to methylating agents used in the clinic [87]. Furthermore, HR induced in healthy tissues during treatment with chemotherapeutic alkylating agents may be linked to therapy-induced secondary cancers [88]. Because of the broad reporter expression and sensitivity to methylation-induced HR, the RaDR-GFP mice offer a new approach for probing the extent to which treatments impact genomic stability both within the tumor and within healthy tissues, which is relevant to the risk of secondary cancers.

In addition to FYDR and RaDR-GFP mice, several other mouse models that harness fluorescence as a marker for HR have been developed, including the HPRTdupGFP, which is currently in development in the Noda laboratory and promises to offer its own advantages. In addition, the Jasin laboratory extended their studies of DSB-induced HR *in vitro* to an animal model. The DR-EGFP mice harbor a recombination reporter that carries sequences for site-specific cleavage by *I-Sce*I, and thus enable studies of DSB-induced HR in cells cultured from that mouse [89]. Using this model, it has been shown that a deficiency in *Brca1* leads to reduced HR in cultured cells, and that DSB-induced HR can be studied in various cell types *in vitro* using cells derived from disaggregated tissues of the DR-EGFP mouse. While the use of a homing endonuclease greatly increases the frequency of HR, making it easier to quantify, the endonuclease needs to be introduced *in vitro*, which is not compatible with studies of HR *in vivo*. Furthermore, the DR-EGFP reporter is integrated into the *Pim-1* locus. In the absence of a positive control, it is not possible to assess the relative frequency of HR among tissues, since a low frequency of fluorescent cells may be due to either a lower rate of HR or suppressed expression of EGFP. In contrast, for the RaDR-GFP mice, it is possible to compare HR among tissues since the number of cells that potentially express EGFP can be deduced using a complementary positive control mouse line with the identical locus and promoter. Unlike the DR-EGFP studies of HR in cells that have been isolated from mice, the mice and the methods described here enable analysis of HR in cells within their normal tissue context *in vivo*, which enables studies of more complex physiological processes, including cancer development and chronic exposures.

Many mouse models have been developed for studies of point mutations/small deletions *in vivo* (*Pig-a*, MutaMouse, Big Blue, Plasmid *lac-z*, *cII*, *Gpt-Δ* [90]–[95]. For each of these mouse models, as well as for the RaDR-GFP mice, susceptibility to sequence changes is being monitored at a specific locus. Although vulnerability to sequence changes is anticipated to be locus dependent, these models nevertheless provide useful tools for assessing the impact of genetic and environmental factors that impinge on genomic stability. Unlike the transgenic models that are used to study point mutations, the RaDR-GFP model exploits fluorescence. The median frequency of fluorescent cells in RaDR-GFP tissues is approximately ∼2/10^5^, whereas the frequency of point mutations is much more rare (∼1/10^8^ per base pair) [1]. Consequently, strategies that exploit fluorescence to detect cells that have undergone a specific point mutation within intact tissue have not yet been described. Success in studies of point mutagenesis has been achieved by isolating DNA from mouse tissues, packaging the DNA into phage particles, and subsequently detecting mutation events via phenotypic change in *E. coli* [91]–[95]. This process is laborious, expensive, slow, and significant expertise is required in order to obtain reliable data, which together severely limit the utility of these models. In contrast, analysis of recombinant cells within intact RaDR-GFP tissue requires minimal expertise, can be performed with standard fluorescent microscopy, and requires much less time (*e.g.*, processing one RaDR-GFP tissue takes minutes, as opposed the many days that are required for analysis of point mutations). Nevertheless, as the underlying factors that modulate point mutagenesis are very different from those that drive HR, methods that enable studies of point mutations and HR are highly complementary.

Intensive research in the past decade has given rise to sophisticated models for the molecular basis of HR, and has revealed that imbalanced HR contributes to genomic instability and cancer [75], [96], [97]. Here, we describe a novel mouse model that enables studies of HR in at least 11 different tissues. Here we show that HR is pervasive among mammalian tissues, that the frequency of HR is tissue-dependent, and that recombination events accumulate with age. The RaDR-GFP mice open doors to a wide range of studies. Knowledge about the extent to which HR is normally active in different tissue types is relevant to our understanding of how defects in HR lead to cancer in certain tissues. By crossing with genetically engineered mice, it is now possible to establish how specific genes impact HR throughout mammalian tissues, and furthermore how HR capacity impinges on cancer development. For example, the HR capacity of tumors that are anticipated to be HR deficient (*e.g.*, those that arise in a *Brca*2+/− mouse model) can potentially be formally tested *in vivo* using the RaDR-GFP model. In terms of exposures, HR can be monitored over time, which makes this model compatible with studies of long-term environmental conditions that are relevant to human cancer risk. Furthermore, this model can serve as a tool in the development of cancer chemotherapeutics by providing a window into tissue specific effects. In particular, the risk of secondary cancers can be reduced by developing approaches that induce HR and associated genotoxicity in the tumor, while suppressing sequence rearrangements in healthy tissues. Additionally, in terms of cancer treatment, the RaDR-GFP mice make it possible to assess the efficacy of pharmaceutical agents that are designed to either suppress or induce HR in a tumor-specific fashion. Taken together, we have demonstrated how key processes, including tissue context, aging and exposure to a DNA damaging agent, impact the risk of HR *in vivo*. By creating new avenues for studies of HR in multiple tissues, the work described here enables future studies of genetic, environmental, and clinical conditions that impact genomic stability in mammals.

## Materials and Methods

### Construction of the RaDR-GFP Substrate

Plasmid construction was described previously [66]. Briefly, truncated EGFP coding sequences (Δ5*egfp* lacking 15 bases at the 5′ end and Δ3*egfp* lacking 81 bases at the 3′ end) were amplified by PCR from plasmid pCX-EGFP, using primers that each insert unique sequences. PCR products were cloned in a tandem orientation (Δ5*egfp* followed by Δ3*egfp*) into the pCX-NNX backbone to form the direct repeat HR substrate, yielding plasmid pCX-NNX-ΔGF. The HR substrate was then cloned into pBigT-TpA, released together with the neomycin resistance gene and cloned into pRosa26PA [68] (a kind gift from Dr. P. Soriano, Mount Sinai School of Medicine) to yield the targeting plasmid pRosa26-ΔGF (Figure S1).

### Creation of RaDR-GFP Transgenic Mouse

All animals were housed and handled in Association for Assessment and Accreditation of Laboratory Animal Care (AAALAC)-accredited facilities with diets, experimental methods, and housing as specifically approved by the Institutional Animal Care and Use Committee. The MIT CAC (IACUC) specifically approved the studies as well as the housing and handling of these animals.

The pRosa26-ΔGF targeting plasmid (Figure S1) was linearized by digestion with *Xho*I (New England Biolabs) and electroporated into mouse 129 embryonic stem (ES) cells. Clones were selected for resistance to G418 by growing in selective media (40% DMEM + glucose, 40% EmbryoMax DMEM, 1% β- mercaptoethanol, 15% FBS, penicillin, streptomycin, glutamine, nonessential amino acids, LIF, G418) and screened for correct targeting by PCR and Southern blot. Cells from clones with correct targeting were injected into the blastocoel of 3.5-day-old C57BL/6 blastocysts, which were implanted into pseudopregnant female mice. All ES cell manipulations and transgenic mouse development were performed by the ES Cell and Transgenics Facility at the Swanson Biotechnology Center of the Koch Institute for Integrative Cancer Research at MIT. All procedures involving mice were approved by the Massachusetts Institute of Technology Committee on Animal Care and in accordance with the National Institutes of Health guidelines for the humane care of animals.

### PCR Analysis

To identify clones with correct targeting of the RaDR-GFP substrate, we used a forward primer annealing 5′ to the targeted locus and a reverse primer landing in the neomycin resistance gene within the construct, yielding a 1.24 kb PCR product (Figure 1C). In the absence of insertion, the forward primer yields a 1.16 kb PCR product with a reverse primer landing within the *Rosa26* locus (Figure 1C). All primer sequences and exact PCR amplification conditions can be found in Tables S1, S2, S3. PCR detection of the Δ5*egfp*, Δ3*egfp*, and full-length *EGFP* sequences was performed as described previously [66].

### RNA Extraction and cDNA Conversion

Embryonic stem (ES) cells (10^4^–10^6^) or RaDR-GFP mouse pancreatic cells (∼1000) were lysed with 1 ml TRIzol (Life Technologies) and either stored at −80°C or processed immediately. Total RNA was extracted and column purified using the RNeasy Mini Kit (Qiagen). Briefly, TRIzol-lysed cells were mixed with 200 µl chloroform and centrifuged at 12,000 g for 15 min at 4°C. The aqueous phase was mixed with 500 µl ice-cold isopropanol and applied to an RNeasy column. The column was washed based on the manufacturer's protocol and RNA was eluted with 30 µl RNase-free water. Total RNA (500–2000 ng) was converted to cDNA with the SuperScript III First-Strand Synthesis System for RT-PCR (Life Technologies) with both random hexamers and oligo(dT). The volume was brought to 10 µl with RNase-free water and incubated at 65°C for 5 min before placing on ice for at least 1 min. Reverse transcriptase master mix was added and the reaction was incubated at 25°C for 10 min, 50°C for 50 min and 85°C for 5 min. Finally, *E.coli* RNase H (1 µl) was added and the reaction was incubated at 37°C for 20 min to remove RNA-cDNA duplexes before proceeding with PCR.

### Direct PCR Analysis Using RNA Transcripts

PCR detection of full-length EGFP sequences was performed with primers A FL FOR and C FL REV using Platinum Taq DNA Polymerase (Life Technologies). 5 µl 10× diluted cDNA was used as the template in the presence of 0.2 µM primers and enzyme mix according to the manufacturer's instructions. cDNA was denatured at 94°C for 3 min, and then incubated for 40 cycles at 94°C for 45 s, 56°C for 45 s and 72°C for 1.5 min. Reactions were then incubated at 72°C for 5 min and placed on ice. In order to detect Δ5*egfp* and Δ3*egfp*, two primer sets were used in a single reaction. Primers E D5 FOR2 and F D5 INT REV were used to detect Δ5*egfp*, and primers G D3 INT FOR and H D3 REV2 were used to detect Δ3*egfp*. Each reaction contained 0.2 µM primers. PCR reactions were incubated at 94°C for 3 min, and then at 94°C for 45 s, 55°C for 30 s and 72°C for 1 min 10 s for 40 cycles. Samples were incubated at 72°C for a final 5 min and placed on ice.

### Nested PCR Analysis for Full-length EGFP

External PCR primers were designed to anneal upstream and downstream of the *EGFP* coding sequence. Primers (0.2 µM) BPEF3 and NEST Rev were added to Platinum Taq DNA polymerase mix with 5 µl 10× diluted cDNA following the manufacturer's protocol. Reactions were incubated at 94°C for 3 min, and then for 40 cycles at 94°C for 45 s, 58°C for 30 s and 72°C for 1 min 10 s. Reactions were ended with incubation at 72°C for 5 min and then placed on ice. PCR products were purified using the MinElute PCR Purification Kit (Qiagen) and eluted with the same volumes of EB buffer. Purified PCR products (5 µl) were used for subsequent full length EGFP PCR as described above. PCR products were analyzed by 1.5% agarose gel electrophoresis.

### Single Cell Nested PCR Analysis

Single cells from RaDR mouse spleen were sorted by FACS into 5 µl lysis buffer (400 ng/µl proteinase K and 17 µM SDS in nuclease-free water). As a control, a single colony of RaDR-GFP ES cells was also added to lysis buffer. Cell lysates were freeze-thawed once at −80°C, and added to a total volume of 50 µl Platinum Taq DNA Polymerase (Life Technologies) mix with 0.2 µM primers BPEF3 and NEST Rev (Table S2). External PCR was performed as described above. External PCR products (2–5 µl) were then used for internal PCR as described above.

### Southern Blot Analysis

The EGFP probing sequence was ^32^P-labeled by random priming (NEBlot, New England Biolabs). Genomic DNA was isolated from candidate clones and digested with *Hin*dIII (New England Biolabs). DNA fragments were resolved by electrophoresis and transferred to a nylon membrane (Hybond-XL, GE Healthcare). The blot was incubated at 65°C in ExpressHyb (BD Biosciences/Clontech) with the ^32^P-labeled EGFP probe. The probed blot was visualized on a Storm 840 PhosphorImager (Molecular Dynamics).

### Positive Control Mouse

B6.Cg-Gt(ROSA)26Sor^tm6(CAG-ZsGreen1)Hze^/J mice (Jackson Laboratory) carry the green fluorescent protein gene ZsGreen1 at the *Rosa26* locus driven by the CAG promoter, with an upstream STOP codon flanked by *loxP* sites and a downstream WPRE mRNA stabilizer. These mice were crossed with B6.C-Tg(CMV-cre)1Cgn/J mice (Jackson Laboratory) that carry the Cre recombinase gene driven by the CMV promoter, resulting in the deletion of *loxP*-flanked sequences in all tissues including the germline. Mice positive for both transgenes were then backcrossed to C57BL/6J. The resulting Cre negative progeny expressing ZsGreen1 under the CAG promoter at the *Rosa26* locus were used to determine the reporter expression profile. Mice were in the C57BL/6 background, and were bred in house. All animals were housed in pathogen free barrier facilities and treated humanely with regard for alleviation of suffering.

### Flow Cytometry

Tissues were kept in 0.01% trypsin inhibitor (Sigma) on ice for up to 16 hours before analysis. Tissues were minced with scalpel blades or with a gentleMACS tissue dissociator (Miltenyi Biotec) and digested with 2 mg/ml collagenase V (Sigma) in HBSS (Invitrogen) at 37°C for 45 min. After digestion, the cell suspension was triturated and filtered through a 70 µm cell strainer (BD Biosciences) into an equal volume of DMEM with 20% FBS on ice. Cells were pelleted at 1500 rpm for 10 minutes, resuspended in OptiMEM (Invitrogen) and passed through a 35 µm cell strainer (BD Biosciences) before flow cytometry. Cells were analyzed with a FACScan flow cytometer (BD Biosciences) or sorted with a MoFlo cell sorter (Cytomation). Live cells were gated using forward and side scatter and then examined for fluorescence (excitation 488 nm, emission 580/30 nm). For RNA extraction from spleen cells, 1000 EGFP positive or 1000 non-EGFP positive cells were sorted into 200 µl TRIzol using a MoFlo (Cytomation) or a FACSAria (BD Biosciences) cell sorter. TRIzol volumes were then made up to 1 ml and cells were stored at −80°C until RNA extraction.

### *In Situ* Imaging of Recombinant Foci and Isolated Crypts

Whole organs were processed for imaging by compressing between coverslips to a thickness of 0.5 mm. The colon was cut lengthwise to expose the lumen. Tissues were imaged with a Nikon 80i microscope (×1 objective) in the FITC channel using a fixed exposure time. Serial images scanning the entire tissue surface were captured using an automated stage. Images were automatically compiled using NIS Elements software (Nikon) or Adobe Photoshop (Adobe Systems). Brightness and contrast of all images were adjusted identically in Adobe Photoshop. Fluorescent foci were either counted manually in a blinded fashion or with an in-house program written in MatLab (MathWorks). Tissue surface area was determined using ImageJ (NIH) by manually tracing the tissue outlines. Frozen sections (5 µm) were imaged with a ×60 objective in the FITC channel, stained with hematoxylin and eosin, and imaged again under visible light. Images were then overlaid manually. For each estimate of the average number of foci per cm^2^, the entire organ was evaluated in order to suppress the impact of variations in foci number in different regions of each organ.

Colonic crypts were isolated according to [98], with some modifications. Briefly, tissue samples were washed with HBSS to remove any fecal material. Dissected samples (0.5 to 1 cm^2^) were treated with 1 mM EDTA, 0.05 mM dithiothreitol (Sigma) at 37°C. After incubation for 30 min, tissue samples were gently shaken in the EDTA/DTT solution by inverting the tubes to release epithelial cells. This process was repeated twice. Crypts were stained with 1 µg/ml Hoechst 33342 (Invitrogen) and imaged with an Axio Observer Z1 microscope (Zeiss) at ×10 in the brightfield, FITC, and DAPI channels. Crypt images were captured using Axiovision Rel. 4.8 software (Zeiss) and compiled with Image J 1.46r (NIH).

### Automated Foci Counting

Images were preprocessed using median filtering, and intensity shoots identified with an extended maxima transform [99] were treated as foci candidates. Candidates were segmented using a local thresholding-based algorithm where the threshold for each focus was adaptively selected by modeling the focus as a two-dimensional Gaussian distribution. Based on intensity and morphological features extracted by preprocessing and segmentation, foci candidates were classified into true foci and noise, and foci were further classified into large bright foci and small irregular foci using a support vector machine (SVM) with a radial basis function (RBF) kernel. The SVM was trained on annotations from an experienced biologist over multiple images.

### DNA Damage-Induced Recombination

Five- to seven-week-old heterozygous RaDR-GFP mice (C57BL/6 background) were used. DNA damage was elicited by combined treatment with N-methyl-N-nitrosourea (MNU, Sigma) and thyroid hormone (T3, Sigma). Details will be published separately. Briefly, T3 was administered in the diet (prepared by TestDiet) at 4 ppm according to [100]. MNU was administered at 25 mg/kg as an intraperitoneal injection at the time of peak cell proliferation in the pancreas induced by T3. Control mice were fed an identical diet without T3, and received control PBS injections. Feeding of T3 continued for 2 days after MNU injection. 3.5 weeks after MNU injection, mice were humanely euthanized and organs were harvested for the RaDR-GFP assay.

### Statistics

Recombinant cell frequencies and foci frequencies do not follow a normal distribution and were therefore compared using a two-tailed Mann–Whitney test. A *p* value of less than 0.05 was considered to be statistically significant.

## Supporting Information

Figure S1 Design strategy for the RaDR-GFP targeting construct.
(JPG)

Figure S2 Single-cell nested PCR analysis to identify recombination classes. (A) Analysis of a single colony of RaDR-GFP ES cells shows the presence of Δ3*egfp* and Δ5*egfp*, but not full-length *EGFP*. (B) Nested PCR analysis of single spleen cells. Tissue was disaggregated and green fluorescent cells were isolated by FACS. In addition to full-length *EGFP*, Δ3*egfp* and/or Δ5*egfp* were also detected, indicating that cells had undergone HR through different pathways (see Figure 3 and text).
(TIF)

Table S1 Sequences for forward and reverse PCR primers that specifically amplify full length *EGFP*, Δ3*egfp* and Δ5*egfp*. Sequences are listed from 5′ to 3′.
(PDF)

Table S2 Sequences for flanking PCR primers used for nested PCR.
(PDF)

Table S3 PCR conditions and product sizes.
(PDF)
