# Supplementary figures and images for: Rosa26-GFP Direct Repeat (RaDR-GFP) Mice Reveal Tissue- and Age-Dependence of Homologous Recombination in Mammals In Vivo
Source: PLoS Genet. 2014 Jun 5;10(6):e1004299. doi: 10.1371/journal.pgen.1004299 (PMC4046920; doi:10.1371/journal.pgen.1004299)

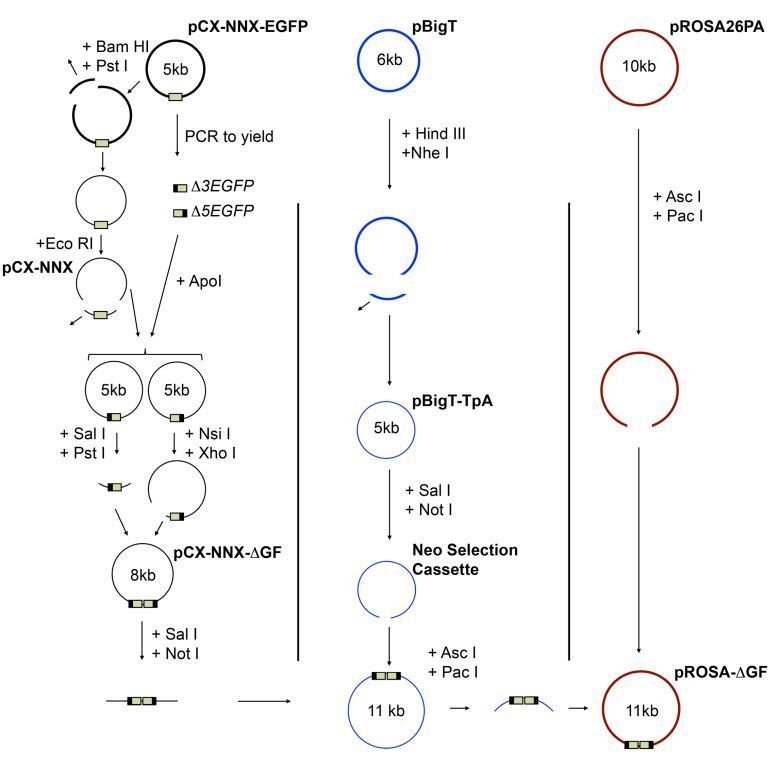

Supplement: Figure S1 — Design strategy for the RaDR-GFP targeting construct. (JPG) [file pgen.1004299.s001.jpg]
