# Supplementary material for: Rosa26-GFP Direct Repeat (RaDR-GFP) Mice Reveal Tissue- and Age-Dependence of Homologous Recombination in Mammals In Vivo
Source: PLoS Genet. 2014 Jun 5;10(6):e1004299. doi: 10.1371/journal.pgen.1004299 (PMC4046920; doi:10.1371/journal.pgen.1004299)
Supplement: Table S1 — Sequences for forward and reverse PCR primers that specifically amplify full length EGFP, Δ3egfp and Δ5egfp. Sequences are listed from 5′ to 3′. (PDF) [file pgen.1004299.s003.pdf]

Supplemental Table 1. Primers for amplification of specific cassettes.

**Gene**	**Direct PCR/Internal PCR primers**	**Primer sequence**
Full length EGFP	A FL FOR	ATT CGC CAC CAT GGT GAG CAA GGG C
	C FL Rev	GAT ATC AAG CTT ACT TGT ACA GCT C
Δ5egfp	E D5 FOR2	ACA GGG TAA CTA GCT GGA TCC
	F D5 INT REV	TGC TTC ATG TGG TCG GGG TAG CGG
Δ3egfp	G D3 INT FOR	TTC TTC AAG TCC GCC ATG CCC GAA
	H D3 REV2	AGA TGC TGA GGT ACC GGA TCC TAT
