# Supplementary material for: Rosa26-GFP Direct Repeat (RaDR-GFP) Mice Reveal Tissue- and Age-Dependence of Homologous Recombination in Mammals In Vivo
Source: PLoS Genet. 2014 Jun 5;10(6):e1004299. doi: 10.1371/journal.pgen.1004299 (PMC4046920; doi:10.1371/journal.pgen.1004299)
Supplement: Table S2 — Sequences for flanking PCR primers used for nested PCR. (PDF) [file pgen.1004299.s004.pdf]

Supplemental Table 2. Primers for nested PCR.

External PCR primers	Primer sequence
BPEF3	CTG ACT GAC CGC GTT ACT CC
Nest Rev	TAG GCA GCC TGC ACC TGA G
