# Supplementary material for: Rosa26-GFP Direct Repeat (RaDR-GFP) Mice Reveal Tissue- and Age-Dependence of Homologous Recombination in Mammals In Vivo
Source: PLoS Genet. 2014 Jun 5;10(6):e1004299. doi: 10.1371/journal.pgen.1004299 (PMC4046920; doi:10.1371/journal.pgen.1004299)
Supplement: Table S3 — PCR conditions and product sizes. (PDF) [file pgen.1004299.s005.pdf]

Supplemental Table 3: Thermal cycler conditions and product sizes.

**Gene**	**Size (bp)**	**PCR conditions**
Full length *EGFP*	740	94ºC, 3 min<br><br>94ºC, 45 s<br>56ºC, 45 s<br>72ºC, 90 s<br>Repeat from step 2, 39x<br><br>72ºC, 5 min<br>4ºC indefinitely
Δ3*egfp*<br>Δ5*egfp*	250<br>415	94ºC, 3 min<br><br>94ºC, 45 s<br>55ºC, 30 s<br>72ºC, 70 s<br>Repeat from step 2, 39x<br><br>72ºC, 5 min<br>4ºC indefinitely
Flanking PCR product for nested PCR	779	94ºC, 3 min<br><br>94ºC, 45 s<br>58ºC, 45 s<br>72ºC, 70 s<br>Repeat from step 2, 39x<br><br>72ºC, 5 min<br>4ºC indefinitely
